# Global research trends and hotspots in mesenchymal stem cell applications for bone regeneration: A bibliometric and visualized analysis

**DOI:** 10.1097/MD.0000000000047904

**Published:** 2026-02-28

**Authors:** Liheng Huang, Lei Li, Fei Xing, Yue Yang, Jiayi Zhu, Rongnan Shi, Qi Deng, Jianxiong Wang, Fuhua Sun

**Affiliations:** a Rehabilitation Medicine Department, The Affiliated Hospital of Southwest Medical University, Luzhou, Sichuan, People’s Republic of China; b Nursing School of Southwest Medical University, Luzhou, Sichuan, People’s Republic of China; c Department of Rehabilitation Medicine, Southwest Medical University, Luzhou, Sichuan, People’s Republic of China; d Rehabilitation Medicine and Engineering Key Laboratory of Luzhou, Luzhou, Sichuan, People’s Republic of China.

**Keywords:** bibliometrics, bone regeneration, CiteSpace, mesenchymal stem cells, scientometrics

## Abstract

**Purpose:**

This study aims to perform a bibliometric analysis of mesenchymal stem cells in relation to bone regeneration from 2005 to 2024. The objective is to identify emerging research hotspots and elucidate collaborative networks within the field.

**Methods:**

Data were extracted from the Web of Science Core Collection on April 20, 2025. Both qualitative and quantitative analyses assessed publication trends, funding sources, journals, authors, institutions, and countries. Visualization and mapping of keywords, collaborations, and co-citation patterns were performed using CiteSpace and Bibliometrix.

**Results:**

A total of 9441 articles were published from 2005 to 2024. Leading journals included *Biomaterials*, *Acta Biomaterialia*, and *Journal of Biomedical Materials Research Part A*. Liu Y emerged as the most prolific author with 167 publications and 5720 citations. China, along with Shanghai Jiao Tong University, ranked first in both national and institutional output. Core collaborations were observed between China, the USA, South Korea, and Germany. Prominent keywords included “osteogenic differentiation,” “mesenchymal stem cells,” “bone regeneration,” “in vitro,” and “scaffolds.”

**Conclusion:**

This study offers a systematic analysis of the literature on mesenchymal stem cells in bone regeneration. *Biomaterials* is identified as the most influential journal in this field, with China, Shanghai Jiao Tong University, and Liu Y leading the research output. Key research hotspots include bone healing, bone metabolism, nanomaterials, and dynamics, while cutting-edge areas such as mitochondrial function, miR-21-5p, cellular senescence, exosomes, extracellular vesicles, and 3D printing are emerging as significant research directions.

## 1. Introduction

Bone defects resulting from aging, severe trauma, and disease remain significant clinical challenges, affecting the functional and psychological well-being of millions of patients.^[1]^ As society ages, these challenges are expected to intensify.^[2]^ Although bone tissue has remarkable self-repair capabilities, severe defects necessitate external interventions to promote regeneration due to insufficient natural healing.^[3]^ Bone grafting has historically been considered the safest and most effective method for bone repair and remains the gold standard for regeneration. However, despite its advantages, bone grafting is hindered by limitations such as prolonged inflammation, donor site morbidity, limited availability, and immune rejection.^[4,5]^ This highlights the urgent need to explore alternative methods to facilitate bone regeneration. In recent years, regenerative medicine has emerged as a promising avenue for bone repair.^[6]^ With the successful approval of stem cell therapies in Europe, regenerative medicine has become a key therapeutic approach.^[7,8]^ Among these therapies, stem cell treatment is pivotal, aiming to enhance the body’s repair mechanisms by modulating endogenous stem cell populations or replenishing the cellular reservoir, thereby promoting tissue homeostasis and regeneration.^[9]^

Mesenchymal stem cells (MSCs), which possess self-renewal and multi-lineage differentiation potential, demonstrate excellent capacity for in vitro expansion and differentiation, making them essential for bone tissue regeneration.^[10,11]^ MSCs play a crucial role in maintaining bone homeostasis and facilitating tissue regeneration,^[12,13]^ Derived from mesodermal adult stem cells, MSCs can differentiate into various cell types, including osteoblasts, chondrocytes, and adipocytes.^[14,15]^ Functioning as a physiological reservoir for tissue defects, MSCs respond to regulatory signals from the injured microenvironment, guiding themselves towards appropriate cell fates. This ability allows MSCs to activate regenerative cascades and modulate the bone microenvironment through the secretion of bioactive factors, such as growth factors and chemokines.^[16–18]^ The success of bone defect repair depends on maintaining MSC physiological functions throughout regeneration and regulating cell fate. In summary, the application of MSCs in regenerative medicine offers a promising therapeutic strategy for bone tissue regeneration,^[19]^ with efficient osteogenic differentiation as a key objective. To help researchers quickly grasp the current state of research, emerging trends, and future directions, a bibliometric analysis is essential.

Bibliometrics is a field of informatics that provides quantitative and qualitative analyses of literature, enabling the assessment of distribution, relationships, and clustering within a research domain.^[20]^ For researchers, bibliometric analysis helps elucidate the latest developments^[21]^ and has become a crucial method for evaluating the credibility, quality, and impact of academic work.^[22,23]^

Currently, tools such as CiteSpace and R’s Bibliometrix have gained popularity for bibliometric analysis and visualization.^[24,25]^ Previous studies have conducted bibliometric analyses related to MSCs and orthopedic diseases. For example, Deng et al^[26]^ analyzed 1489 articles on the application of MSCs in orthopedics published between 2002 and 2021, identifying key research trends across categories like country, institution, journal, author, and keywords. Similarly, Wang et al^[27]^ reviewed 2100 articles published between 2012 and 2021, exploring research hotspots related to MSCs in osteoporosis and forecasting future trends regarding MSC mechanisms and their role in osteoporosis. Despite the growing body of research on MSCs in bone regeneration, comprehensive bibliometric studies in this domain remain limited. Therefore, we conducted a descriptive statistical analysis of literature on MSCs for bone regeneration from 2005 to 2024, using the Web of Science Core Collection (WOSCC). We also employed CiteSpace and Bibliometrix to examine the evolution of research outputs, collaboration patterns, hotspots, and emerging trends, providing valuable insights for researchers in the field.

## 2. Materials and methods

The WOSCC hosts an extensive array of high-quality, impactful scientific studies, with over 12 million articles available for bibliometric analysis,^[28]^ making it the most frequently used database in this field.^[29]^ On April 20, 2025, we searched the WOSCC (SCI-EXPANDED) database for articles related to the application of MSCs in bone regeneration. The search query was set as follows: [TI=(“mesenchymal stem cell” OR “mesenchymal stem cells”) AND (“bone regeneration” OR “bone repair” OR “bone tissue engineering” OR “osteogenesis” OR “bone reconstruction” OR “bone healing”)] OR [AB=(“mesenchymal stem cell” OR “mesenchymal stem cells”) AND (“bone regeneration” OR “bone repair” OR “bone tissue engineering” OR “osteogenesis” OR “bone reconstruction” OR “bone healing”)] OR [AK=(“mesenchymal stem cell” OR “mesenchymal stem cells”) AND (“bone regeneration” OR “bone repair” OR “bone tissue engineering” OR “osteogenesis” OR “bone reconstruction” OR “bone healing”)]. The search covered literature published from January 1, 2005 to December 31, 2024, including both articles and reviews, and was limited to publications in English.

We initially gathered general information and citation data by reviewing the literature in the WOSCC database. Along with counting the number of articles, citations, and citation density, we employed 2 composite metrics (the H-index and G-index) to assess the impact of authors, journals, institutions, and countries. These metrics reflect both the quantity and quality of research output, with the H-index accounting for both the number of papers and their citation frequency.^[25]^

**Figure 1.**
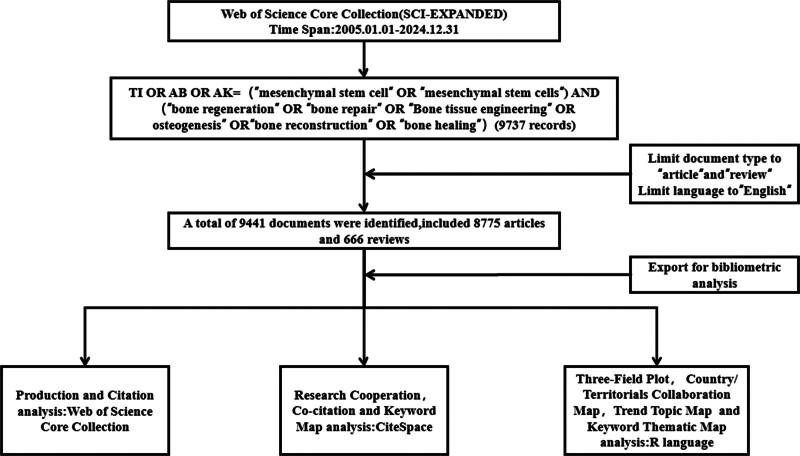
The flow chart illustrating the search strategy and analysis process.

Subsequently, we analyzed scientific collaborations among authors, institutions, and countries, along with their interconnections, using CiteSpace version 6.2.R6 (Drexel University, Philadelphia) and R software (The R Foundation for Statistical Computing, Vienna, Austria). We also examined cited references, authors, and journals. To identify emerging research hotspots and trends, we employed Keyword Co-occurrence Analysis in CiteSpace, along with Trend Topic Mapping and Keyword Thematic Mapping in R. A flowchart illustrating the literature search and analysis process is shown in Figure 1.

In the CiteSpace analysis, the time frame from 2005 to 2024 was divided into annual slices. We considered various node types, such as authors, institutions, countries/regions, keywords, references, cited authors, and cited journals, selecting one node type for analysis at a time. Cluster network analyses for co-cited literature, authors, journals, and keywords were also conducted. The *Q*-value of the clustering network measured the degree of modularity, with values exceeding 0.3 indicating significant clustering. Higher *Q*-values reflected a more pronounced clustering phenomenon. We also used the *S*-value to assess the tightness and separateness of the clustering network, with an *S*-value >0.7 indicating high-quality clustering, and values closer to 1 denoting greater network tightness and separateness.

R software, integrated with statistical analysis and visualization capabilities,^[30]^ was employed to process and analyze datasets retrieved from the WOSCC database using the Bibliometrix package in RStudio (version 4.4.1; Posit Software, PBC, Boston). Through Biblioshiny, we generated several visualizations, including a 3-fields plot, Countries/Regions Collaboration Map, Trend Topic Map, and Keyword Thematic Map. The 3-fields plot presents overarching statistics and relationships or correlations between key elements of scientific collaboration. The Countries/Regions Collaboration Map illustrates the collaborative efforts among different nations within the research domain. The Trend Topic Map tracks the frequency of specific keywords in the literature over time, providing insights into evolving trends. The Keyword Thematic Map categorizes research themes into 4 quadrants: the top left quadrant represents niche themes, which exhibit high density but low centrality, indicating specific but isolated themes; the top right quadrant encompasses motor themes, which display both high density and centrality, marking them as core and well-developed topics. The bottom left quadrant denotes emerging or declining themes, characterized by low density and centrality, which are nascent or marginal topics. Finally, the bottom right quadrant is dedicated to basic themes, which have low density but high centrality, indicating foundational topics with potential to evolve into future research hotspots.

## 3. Results

### 3.1. Publication outputs

**Figure 2.**
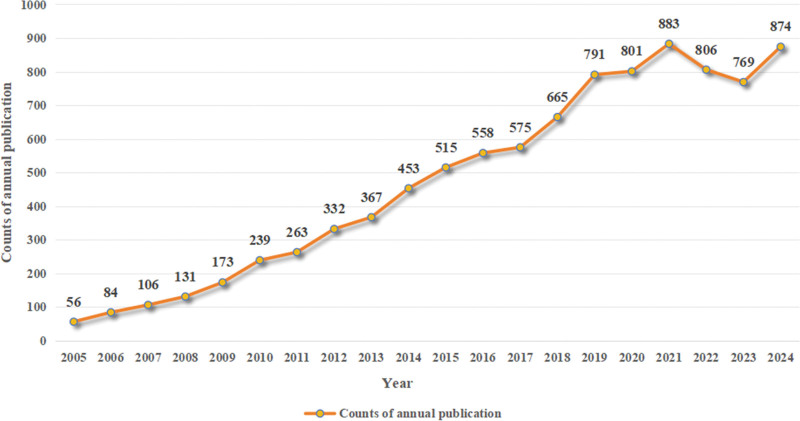
The annual number of publications from 2005 to 2024.

Following the establishment of the search strategy and time frame, a total of 9737 articles related to the application of MSCs in bone regeneration were identified. After restricting the search to English-language articles, including research papers and reviews, 9441 articles remained, consisting of 8775 research papers and 666 reviews, with an average citation rate of 36.97 per article. Figure 2 illustrates the annual publication volume, divided into 3 phases: 2005 to 2011, 2011 to 2021, and 2021 to 2024.

In the 1st phase, publication growth was relatively slow, with an average annual increase of approximately 34 articles, rising from 56 in 2005 to 263 in 2011. The 2nd phase saw a marked increase, averaging 62 additional articles per year. Notably, between 2017 and 2019, the volume surged by 216 articles. By 2021, publications peaked at 883 articles. The 3rd phase experienced fluctuations, with a decline in publications in 2022 and 2023; however, there was a resurgence in 2024, reaching 874 articles.

A total of 8663 funding sources supported research in this area, with the top 10 grant-making organizations contributing to 6291 articles. Table 1 lists the leading funding sources, with the National Natural Science Foundation of China, the National Institutes of Health in the USA, and the United States Department of Health and Human Services ranking as the top 3.

**Table 1:** The top 10 funding sources.

Ranking	Funding sources	Country	Frequence
1	National Natural Science Foundation of China (NSFC)	China	3025
2	National Institutes of Health (NIH) USA	USA	800
3	United States Department of Health Human Services	USA	800
4	National Key Research and Development Program of China	China	321
5	Ministry of Education Culture Sports Science and Technology Japan Mext	Japan	241
6	China Postdoctoral Science Foundation	China	238
7	Fundamental Research Funds for The Central Universities	China	228
8	Japan Society for The Promotion of Science	Japan	226
9	Grants in Aid for Scientific Research Kakenhi	Japan	210
10	European Union (EU)	European Union	202

### 3.2. Journals

From 2005 to 2024, a total of 1089 journals published articles related to MSCs and bone regeneration. The top 10 journals, listed in Table 2, accounted for approximately 18.8% of total publications. According to Bradford Law, these journals form the core publications in this research domain.^[31]^ *Biomaterials* was the most prolific journal, publishing 256 articles, followed by *Acta Biomaterialia* with 209 articles, and *Journal of Biomedical Materials Research Part A* with 198 articles.

**Table 2:** The top 10 journals with the most published literature from 2005 to 2024.

Ranking	Journal	Publications	citation	Citation (per article)	Citation density	H-index	G-index	IF (2023)	JCR
1	*Biomaterials*	256	29,950	116.99	22.5	102	161	12.822	ENGINEERING, BIOMEDICAL (Q1); MATERIALS SCIENCE, BIOMATERIALS (Q1)
2	*Acta Biomaterialia*	209	13,465	64.43	15.1	69	103	9.430	ENGINEERING, BIOMEDICAL (Q1); MATERIALS SCIENCE, BIOMATERIALS (Q1)
3	*Journal of Biomedical Materials Research Part A*	198	6984	35.27	5.9	47	66	3.917	ENGINEERING, BIOMEDICAL (Q2); MATERIALS SCIENCE, BIOMATERIALS (Q2)
4	*Tissue Engineering Part A*	181	7703	42.56	6.2	52	71	3.512	CELL & TISSUE (Q3); CELL BIOLOGY (Q3); ENGINEERING, BIOMEDICAL (Q2); MATERIALS SCIENCE, BIOMATERIALS (Q3)
5	*Stem Cell Research Therapy*	177	8916	50.37	14.7	53	86	7.100	CELL & TISSUE ENGINEERING (Q4); BIOTECHNOLOGY & APPLIED MICROBIOLOGY (Q4); CELL BIOLOGY (Q4)
6	*International Journal of Molecular Sciences*	175	4891	27.95	11.0	38	61	4.862	BIOCHEMISTRY & MOLECULAR BIOLOGY (Q1); CHEMISTRY, MULTIDISCIPLINARY (Q2)
7	*ACS Applied Materials Interfaces*	164	8440	51.46	13.1	55	85	8.300	MATERIALS SCIENCE, MULTIDISCIPLINARY (Q1); NANOSCIENCE & NANOTECHNOLOGY (Q2)
8	*Scientific Reports*	148	5655	38.21	9.5	42	67	3.834	MULTIDISCIPLINARY SCIENCES (Q2)
9	*Journal of Materials Chemistry B*	145	4298	29.64	6.6	36	55	6.119	MATERIALS SCIENCE, BIOMATERIALS (Q1)
10	*Journal of Tissue Engineering and Regenerative Medicine*	125	3389	27.11	4.8	33	48	3.065	BIOTECHNOLOGY & APPLIED MICROBIOLOGY (Q2); CELL & TISSUE ENGINEERING(Q3); CELL BIOLOGY (Q3); ENGINEERING, BIOMEDICAL (Q2)

JCR = Journal Citation Reports.

Among the top 10 journals, *Biomaterials* holds the highest impact factor (IF) of 12.822, followed by *Acta Biomaterialia* (IF 9.430) and *ACS Applied Materials & Interfaces* (IF 8.300). All 10 journals have an IF exceeding 3.0. According to the Journal Citation Reports, these 3 journals are in the Q1 category: *Biomaterials* (ENGINEERING, BIOMEDICAL: Q1; MATERIALS SCIENCE, BIOMATERIALS: Q1), *Acta Biomaterialia* (ENGINEERING, BIOMEDICAL: Q1; MATERIALS SCIENCE, BIOMATERIALS: Q1); and *ACS Applied Materials Interfaces* (MATERIALS SCIENCE, MULTIDISCIPLINARY: Q1).

In terms of citations, articles published in *Biomaterials*, *Acta Biomaterialia*, *ACS Applied Materials & Interfaces*, and *Stem Cell Research & Therapy* have averaged over 50 citations, with *Biomaterials* leading at 116 citations per article. The H-index and G-index for each journal are detailed in Table 2. *Biomaterials* has the highest H-index (102) and G-index (161), followed by *Acta Biomaterialia* (H-index 69, G-index 103). *ACS Applied Materials & Interfaces* has an H-index of 55, while *Stem Cell Research & Therapy* ranks third in G-index (83), securing the third position in both H-index and G-index rankings.

### 3.3. Authors

From 2005 to 2024, a total of 29,255 authors contributed to the literature on MSCs and bone regeneration. The top 10 authors with the highest number of publications are listed in Table 3. The leading authors are Liu Y from China Medical University (167 articles), Zhang Y from Shenzhen University (141 articles), and Wang Y from Shanghai Jiao Tong University (130 articles). Wang Y, ranking third in publication volume, has the highest citation impact, with an average of 50.49 citations per article. Additionally, Wang holds the highest H-index and G-index, with values of 40 and 78, respectively.

**Table 3:** The top 10 active authors with the most publications from 2005 to 2024.

Ranking	Authors	Institution	Publications	Citation	Citation (per article)	Citation density	H-index	G-index
1	Liu Y	China Medical University	167	5720	34.25	10.2	39	68
2	Zhang Y	Shenzhen University	141	3893	27.61	8.6	35	56
3	Wang Y	Shanghai Jiao Tong University	130	6564	50.49	13.2	40	78
4	Wang J	Tongji University	120	3639	30.33	8.2	35	57
5	Wang L	Beihang University	109	4002	36.72	9.9	33	60
6	Li Y	China University of Geosciences(Wuhan)	109	3144	28.84	7.8	33	50
7	Wang H	Chongqing Medical University	105	3181	30.30	8.3	34	53
8	Li J	Shanghai Jiao Tong University	84	2331	27.75	6.9	26	50
9	Zhang X	University of Science & Technology of China	99	4191	42.33	10.2	30	63
10	Liu J	Sichuan University	86	2512	29.21	5.7	26	47

**Figure 3.**
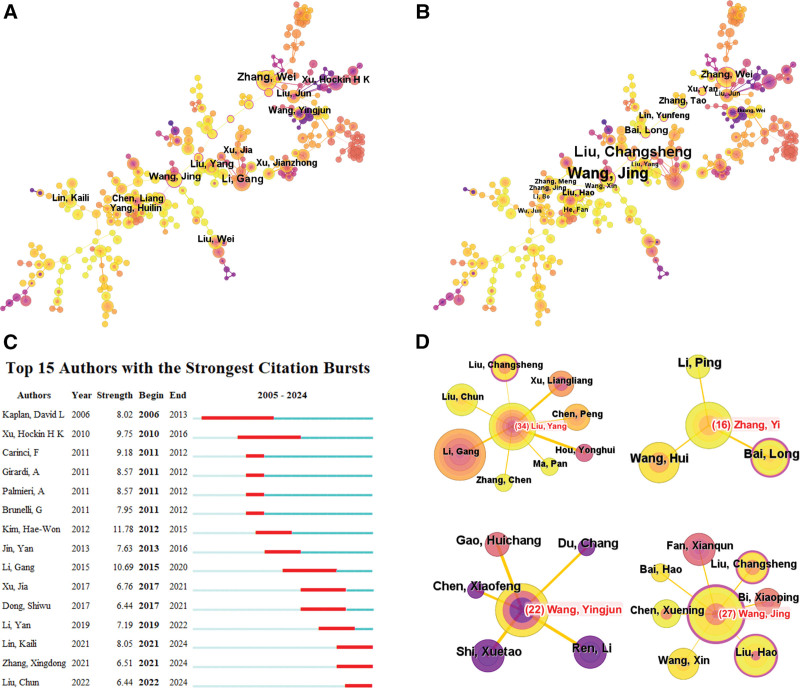
Co-occurrence analysis of authors. (A) Top 10 authors in terms of co-occurrence counts. (B) Top 10 authors in terms of centrality. (C) Top authors with strongest bursts. (D) Collaboration network of some top authors.

Figure 3 illustrates the largest coauthor subnetworks generated using CiteSpace. Based on co-occurrences, the top 3 authors are Zhang Wei (49), Li Gang (36), and Liu Yang (24) (Fig. 3A). In terms of centrality, the top 3 authors are Wang Jing, Liu Changsheng, and Zhang Wei (Fig. 3B). Liu Yang, the author with the highest publication volume, has engaged in extensive collaborations with several other authors, including Li Gang, Liu Changsheng, Liu Chun, and Xu Liangliang (Fig. 3D). Figure 3C shows the top 15 authors with the most significant bursts, indicating a sharp increase in their publications during specific periods. The top 3 authors demonstrating the strongest publication bursts are Kim Hae-Won (2012–2015), Li Gang (2015–2020), and Xu Hockin H. K. (2010–2016).

### 3.4. Institutions

Between 2005 and 2024, a total of 5274 organizations contributed to MSC research for bone regeneration. The top 10 institutions based on publication volume are listed in Table 4. Shanghai Jiao Tong University leads with 562 articles, followed by Sichuan University (370 articles) and the Chinese Academy of Sciences (287 articles). The remaining 7 institutions have more modest outputs, averaging approximately 195 publications each.

**Table 4:** The top 10 institutions with the most publications from 2005 to 2024.

Ranking	Institution	Countries	Publications	Citation	Citation (per article)	Citation density	H-index
1	Shanghai Jiao Tong University	China	562	22,682	40.36	17.7	72
2	Sichuan University	China	370	12,002	32.44	13.4	58
3	Chinese Academy of Sciences	China	287	14,144	49.28	17.9	62
4	Zhejiang University	China	236	8008	33.93	11.2	49
5	Peking University	China	225	8147	36.21	13.8	52
6	Air Force Military Medical University	China	200	8230	41.15	11.3	52
7	Southern Medical University China	China	196	5048	25.76	9.5	40
8	Sun Yat Sen University	China	174	5519	31.72	9.2	41
9	Nanjing Medical University	China	172	4253	24.73	8.8	37
10	University of California System	USA	166	9624	57.98	12.3	53

In terms of H-index, the leading institutions are Shanghai Jiao Tong University (72), the Chinese Academy of Sciences (62), and Sichuan University (58). Notably, the University of California System has the highest average citation count per article, with approximately 58 citations on average.

**Figure 4.**
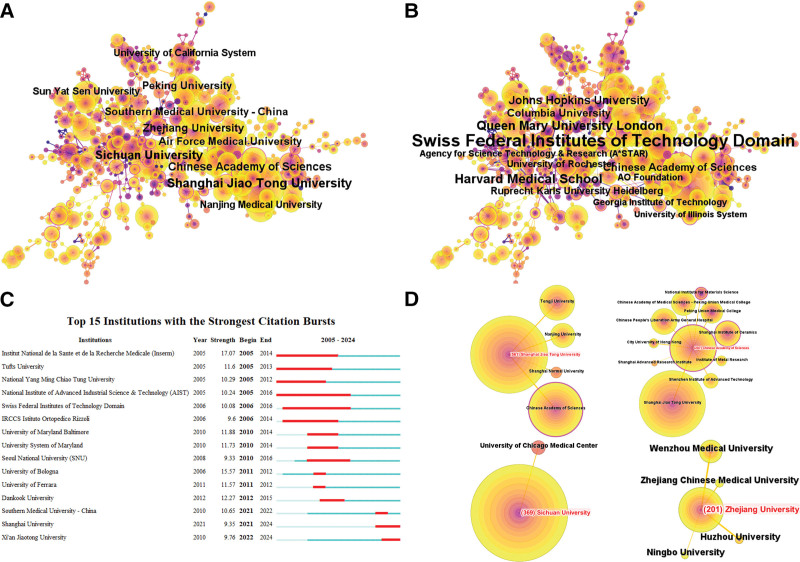
Co-occurrence map of institutions. (A) Top 10 institutions in citation counts. (B) Top 10 institutions in centrality. (C) Top institutions with strongest bursts. (D) Collaboration network of partial institutions.

Figure 4A shows the top 10 institutions by co-occurrence, with Shanghai Jiao Tong University, Sichuan University, and the Chinese Academy of Sciences taking the top 3 positions. These institutions maintain strong collaborative ties, as shown in Figure 4D. For example, the Chinese Academy of Sciences, Nanjing University, Shanghai Normal University, and Tongji University frequently collaborate with Shanghai Jiao Tong University. Furthermore, the Chinese Academy of Sciences has established partnerships with 10 institutions, including the Shanghai Institute of Ceramics, the Chinese Academy of Medical Sciences-Peking Union Medical College, and the Chinese People’s Liberation Army General Hospital. In contrast, Sichuan University, which ranks second in publication volume and third in co-occurrence, has a relatively limited collaboration network.

Figure 4B highlights the institutions with the highest centrality scores, with the Swiss Federal Institutes of Technology, Harvard Medical School, and Queen Mary University of London leading the rankings. Figure 4C depicts the institutions with the most significant bursts in publication volume. The top 3 institutions are the Institut National de la Santé et de la Recherche Médicale (Inserm) (2006–2014), the University of Bologna (2011–2012), and Dankook University (2012–2015).

### 3.5. Countries/regions

Table 5 presents the top 10 countries/regions based on publications, citations, citation density, and H-index. China, the USA, and South Korea are the leading countries, contributing 4635, 1706, and 537 articles, respectively. Notably, the top 2 countries account for approximately 67% of the total publications. Among these countries, the USA has the highest H-index (137) and the highest citations per article (58.19), with the top 6 countries all having an H-index exceeding 60. Although China ranks second in H-index, it has the lowest citations per article.

**Table 5:** The top 10 countries/regions with the most publications from 2005 to 2024.

Ranking	Countries/regions	Publications	Citation	Citation (per article)	Citation density	H-index
1	China	4635	139,777	30.16	29.3	134
2	USA	1706	99,273	58.19	49.1	137
3	South Korea	537	20,701	38.55	15.0	70
4	Germany	469	23,516	50.14	17.5	81
5	Japan	421	17,040	40.48	12.8	66
6	Italy	390	15,659	40.15	13.8	68
7	Iran	368	9226	25.07	9.5	51
8	England	304	17,605	57.91	13.6	70
9	Taiwan	251	10,788	42.98	10.4	59
10	India	243	8766	36.07	11.1	50

**Figure 5.**
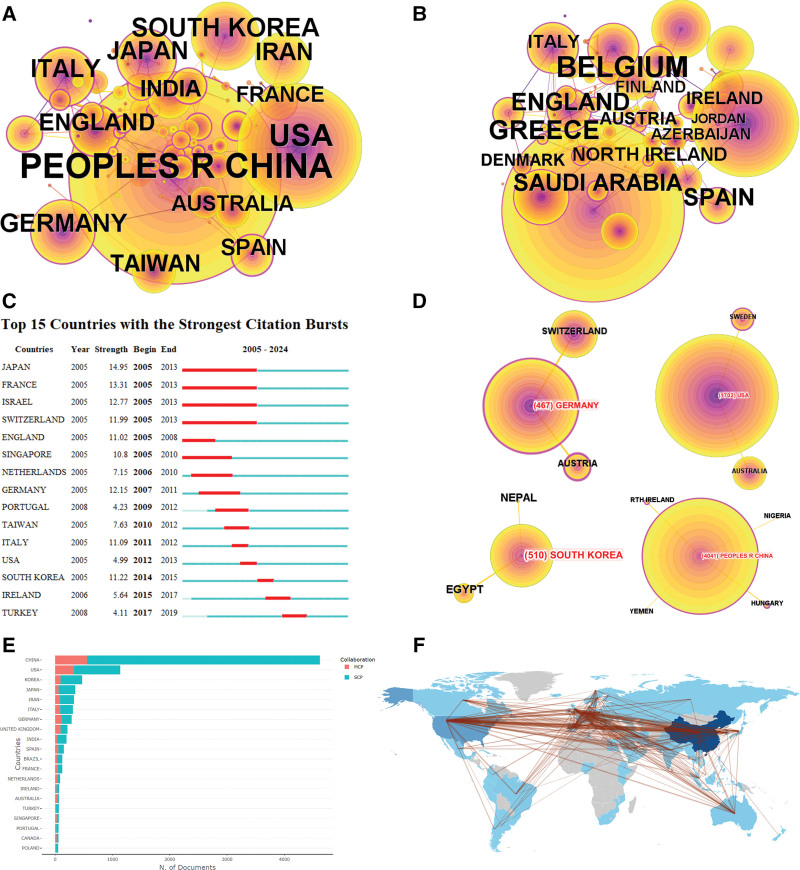
Co-occurrence analysis of countries/regions. (A) Top 10 countries/territories in terms of co-occurrence counts. (B) Top 10 countries/regions in terms of centrality. (C) Top countries/regions with strongest bursts. (D) Cooperation network of some countries/regions. (E) Cooperation network of some corresponding authors. (F) Countries/regions collaboration map.

Figure 5 illustrates the collaborative network, with China (4633), the USA (1702), and South Korea (537) being the top 3 countries by co-occurrence (Fig. 5A). In terms of centrality, Belgium, Greece, and England are the most prominent countries (Fig. 5B). Figure 5C depicts the countries with the most rapid growth in publication numbers, with Japan (2005–2013), France (2005–2013), and Israel (2005–2013) leading. Figure 5D and F highlight international collaborations, showing that China collaborates closely with Hungary, Nigeria, Yemen, and Northern Ireland, while the USA has strong partnerships with Sweden and Australia.

Figure 5E indicates that the top 3 countries with the highest number of internationally collaborative articles are China (MCP: 556), the USA (MCP: 326), and Germany (MCP: 118). Among the top 10 countries by publication volume, the countries with the highest proportions of internationally collaborative articles are England (MCP proportion: 44.2%), Germany (MCP proportion: 41.0%), and Spain (MCP proportion: 36.8%). China’s main collaborators include the USA (425 collaborations), Australia (68 collaborations), and Germany (60 collaborations). The USA’s key collaborators are South Korea (73 collaborations), Germany (60 collaborations), and England (47 collaborations). Furthermore, countries with high publication volumes, such as China and the USA, also have the largest number of articles authored solely by domestic researchers.

### 3.6. Co-citation analysis

Co-citation analysis provides researchers with crucial references and decision-making support by elucidating the structure and dynamics of academic research. The most frequently co-cited literature forms a foundational basis for related fields of study. Table 6 lists the 10 most co-cited articles, 3 of which are indexed in leading journals, primarily focusing on the mechanisms of signal transduction and the regulation of MSCs within the local microenvironment to enhance osteogenic differentiation. Citation bursts refer to articles that experience a sharp increase in citations over a specific period, highlighting significant advancements in the field. Such citation bursts often identify emerging research frontiers and can predict future trends.

**Table 6:** The top 10 reference with most co-citation counts from 2005 to 2024.

Ranking	Title	First author	Journal	IF (2023)	Frequency	Publication year
1	Fate decision of mesenchymal stem cells: adipocytes or osteoblasts?	Q Chen	*Cell Death and Differentiation*	13.658	183	2016
2	The control of human mesenchymal cell differentiation using nanoscale symmetry and disorder	Matthew J Dalby	*Nature Materials*	37.201	174	2007
3	Geometric cues for directing the differentiation of mesenchymal stem cells	Kristopher A Kilian	*Proceedings of the National Academy of Sciences of the United States of America*	9.423	167	2010
4	Coupling of angiogenesis and osteogenesis by a specific vessel subtype in bone	Anjali P Kusumbe	*Nature*	50.484	162	2014
5	Mesenchymal stem cell-macrophage crosstalk and bone healing	Jukka Pajarinen	*Biomaterials*	12.822	131	2019
6	TGF-β and BMP signaling in osteoblast, skeletal development, and bone formation, homeostasis and disease	Mengrui Wu	*Bone Research*	14.312	125	2016
7	The biology of fracture healing	Richard Marsell	*Injury-International Journal of the Care of the Injured*	2.164	106	2011
8	Inflammation, fracture and bone repair	Florence Loi	*Bone*	3.545	102	2016
9	Mesenchymal stem cell-based tissue regeneration is governed by recipient T lymphocytes via IFN-γ and TNF-α	Yi Liu	*Nature Medicine*	58.720	97	2011
10	Osteogenic differentiation of mesenchymal stem cells is regulated by osteocyte and osteoblast cells in a simplified bone niche	E Birmingham	*European Cells & Materials*	3.200	95	2012

TGF-β = transforming growth factor-β, TNF-α = tumor necrosis factor-α.

The most co-cited article is “Fate Decision of Mesenchymal Stem Cells: Adipocytes or Osteoblasts?” by Rowena McBeath et al, published in *Cell Death and Differentiation*. This study, analyzing multiple research findings, establishes a comprehensive system to explore MSCs’ roles, including factors like Runt-related transcription factor 2 (RUNX2), PDZ-binding motif, and the extracellular matrix. However, current research remains in its early stages, underscoring the need for further in-depth studies to fully elucidate the mechanisms balancing lipogenic and osteogenic differentiation of MSCs, which could lead to novel therapeutic approaches for advancing MSC applications in tissue engineering and regenerative medicine.^[32]^

Another significant article is “The Control of Human Mesenchymal Cell Differentiation Using Nanoscale Symmetry and Disorder,” published in Nature Materials by Matthew J. Dalby et al. This study demonstrates how nanoscale disordered stimulation of MSCs induces bone mineral production, addressing limitations of traditional implant materials that often fail due to encapsulation in soft tissue.^[33]^

**Figure 6.**
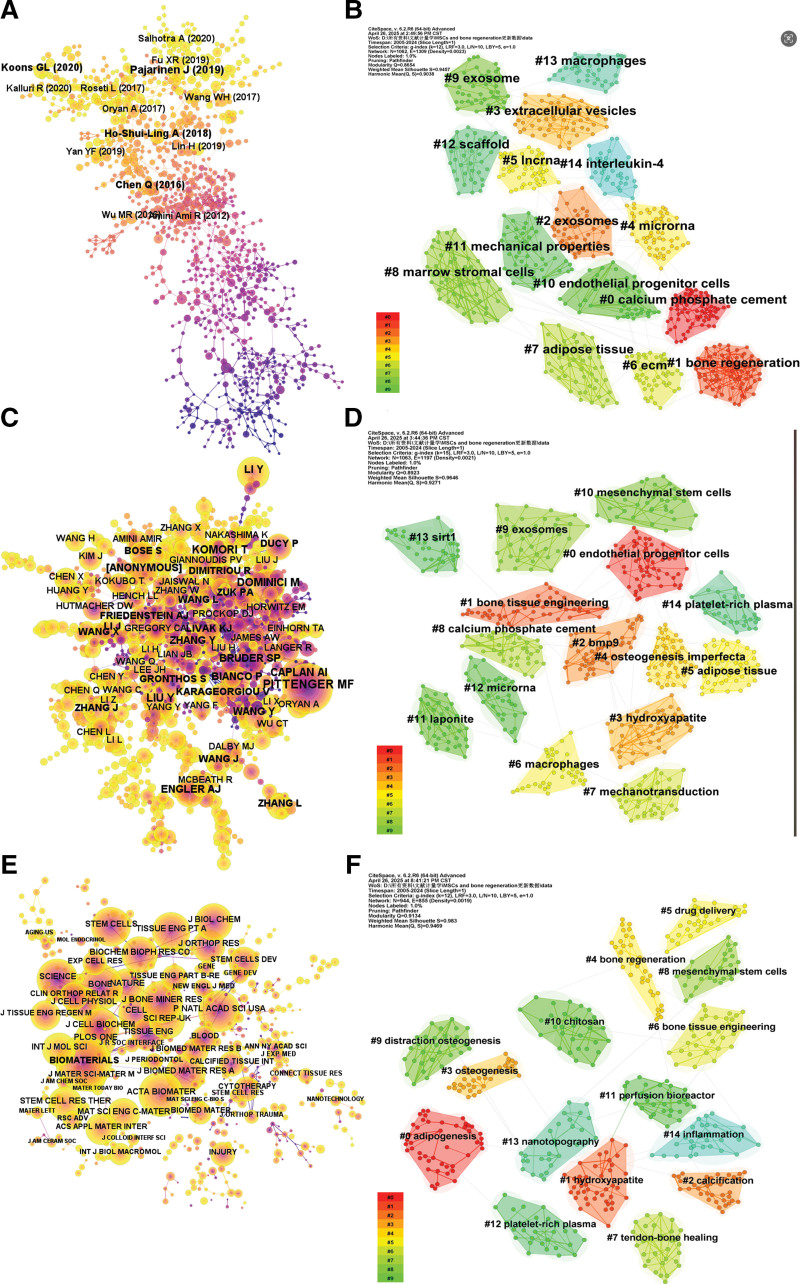
Co-citation analysis of references, authors, and journals. (A) Top 10 references in co-citation counts. (B) A clustering map of the co-citation network of references. (C) Top 10 authors in co-citation counts. (D) A clustering map of the co-citation network of authors. (E) Top 10 journals in co-citation counts. (F) A clustering map of the co-citation network of journals.

The co-citation cluster diagram comprises 15 clusters, with a *Q*-value of 0.8654 and an *S*-value of 0.9038. Figure 6A and B displays the maximum subnet map and the co-cited reference cluster map, respectively. The research base from 2005 to 2024 includes topics such as calcium phosphate cements, bone regeneration, exosomes, extracellular vesicles (EVs), microRNA (miRNAs), long noncoding RNA (lncRNA), extracellular matrix, adipose tissue, and marrow stromal cells. Figure 6C and D depicts the largest subnetworks and clusters of co-cited authors. The top 3 most co-cited authors are Pittenger MF (1271 citations), Caplan AI (649), and Dominici M (642), all of whom are foundational figures in MSC and bone regeneration research.

Figure 6E and F shows the largest subgraphs and clusters of co-cited journals. The top 3 journals ranked by co-citation count are *Biomaterials*, *Bone*, and *Acta Biomaterialia*. Notably, there is a 30% overlap between the top 10 journals by publication volume and those by co-citation count, indicating that articles from these leading journals form a significant foundation for related research.

### 3.7. Keywords and topic terms

**Figure 7.**
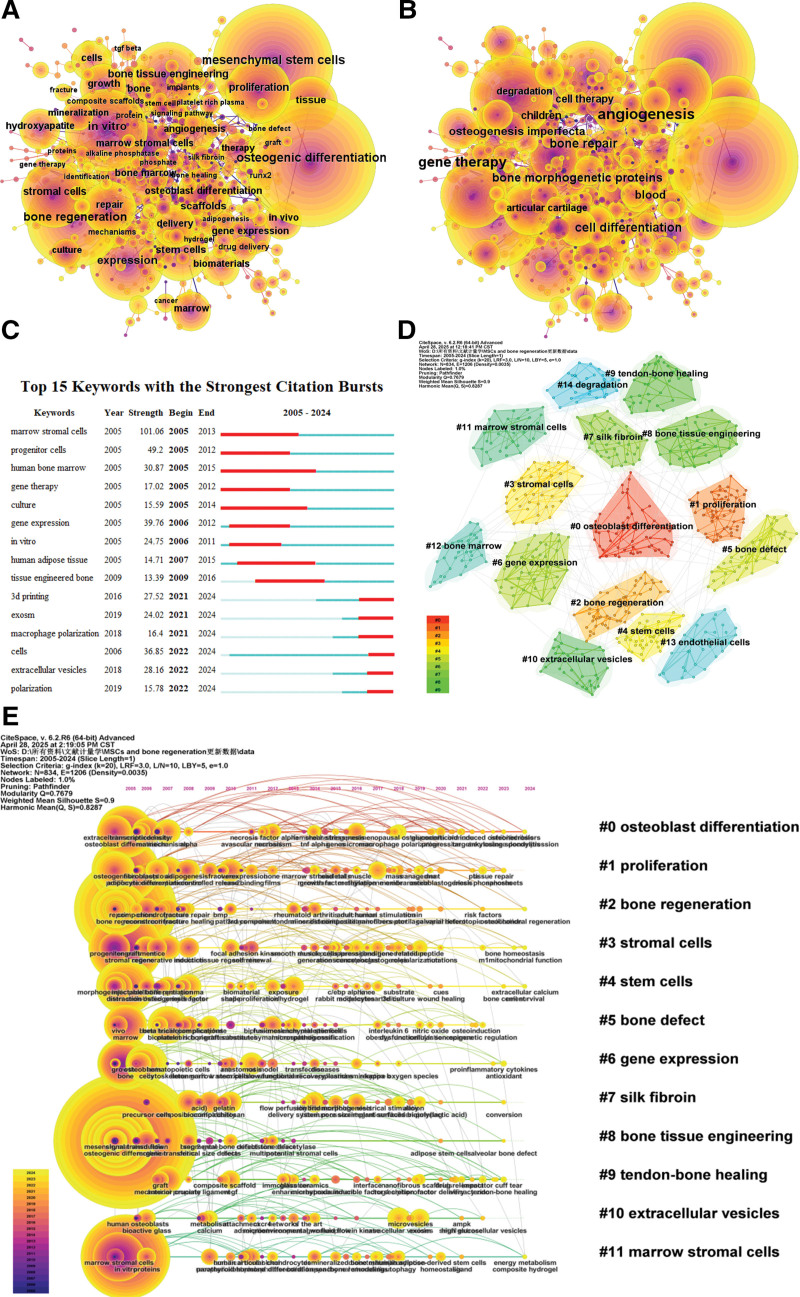
Co-occurrence map of keywords. (A) Top 10 keywords in citation counts. (B) Top 10 keywords in centrality. (C) Top keywords with strongest bursts. (D) A clustering map of the co-citation network of keywords. (E) Keyword timeline visualization from 2005 to 2024 generated by CiteSpace.

Figure 7A presents a network visualization of the keywords, with node size reflecting keyword frequency. The most common keywords include: osteogenic differentiation (4189), mesenchymal stem cells (3627), bone regeneration (2248), in vitro (1746), expression (1425), bone tissue engineering (BTE) (1292), stromal cells (1169), proliferation (1107), scaffolds (916), and stem cells (831). The top 3 keywords with the highest centrality are angiogenesis, gene therapy, and bone repair (Fig. 7B).

Figure 7C highlights the top 15 keywords exhibiting the strongest bursts, with the leading 3 being marrow stromal cells (2005–2013), progenitor cells (2005–2012), and gene expression (2006–2012). Keywords that are currently experiencing citation bursts include: cells (36.85), EVs (28.16), 3D printing (27.52), exosomes (24.02), macrophage polarization (16.40), and polarization (15.78).

In the keyword clustering analysis (Fig. 7D), we identified 15 clusters within the co-word network, each denoted by an ID number (#0, #1, #2, etc). Larger clusters contain more members, with a *Q*-value of 0.7679 and an *S*-value of 0.8287. The categorized keywords span osteoblast differentiation, proliferation, bone regeneration, stromal cells, stem cells, bone defect, gene expression, silk fibroin, BET, tendon-bone healing, EVs, marrow stromal cells, bone marrow, endothelial cells, and degradation.

CiteSpace generated a network comprising 834 nodes and 1206 connections, with a density of 0.0035 (Fig. 7E). This network illustrates the research hotspots and developmental trajectories related to MSC applications in bone regeneration over time. Keywords prevalent from 2005 to 2007 included osteogenic differentiation, mesenchymal stem cells, stromal cells, stem cells, bone regeneration, in vitro, BET, repair, proliferation, scaffolds, osteoblasts, growth factors, matrix, delivery, biomaterials, osteoporosis, and mechanical properties. In contrast, keywords from 2022 to 2024 have shifted towards areas like phosphorus deficiency, health, zinc, muscle, tendon-bone healing, antioxidants, nanosheets, catenin, nanomaterials, mitochondrial function, injectable hydrogels, dynamics, bone homeostasis, and bone metabolism.

**Figure 8.**
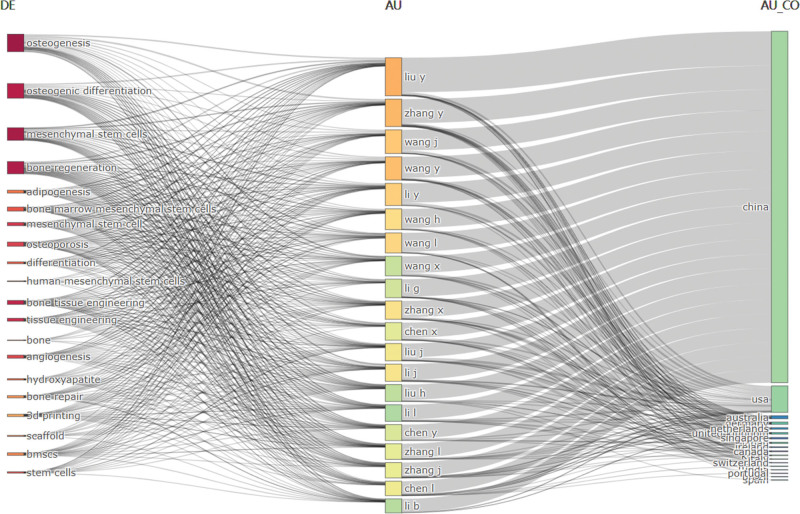
Relations between keywords (left), authors (middle) and countries (right) for research.

**Figure 9.**
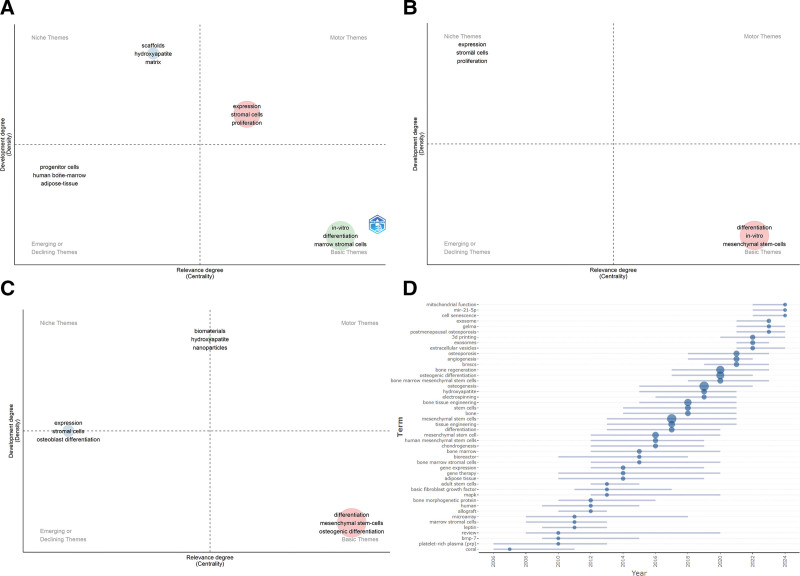
The keyword thematic map. (A) Keyword thematic map from 2005 to 2001. (B) Keyword thematic map from 2011 to 2021. (C) Keyword thematic map from 2021 to 2024. (D) The change trends in the topic terms.

The thematic frequency plot (Fig. 8) illustrates the relationships among keywords, authors, and leading countries in publications. Figure 9A–C present the Keyword Thematic Map, which divides research theme evolution into 3 periods, marked by 2011 and 2021. From 2005 to 2011, keywords such as progenitor cells, human bone marrow, and adipose tissue showed limited developmental prospects, while expression, stromal cells, and proliferation were significant and flourishing in the first quadrant. Between 2011 and 2021, promising keywords included differentiation, in vitro, and mesenchymal stem cells. From 2021 to 2024, differentiation and mesenchymal stem cells emerged as key areas with strong development potential.

Recent years (2011–2024) have seen an increase in topics such as mitochondrial function, miR-21-5p, cell senescence, exosomes, GelMA, 3D printing, angiogenesis, and EVs (Fig. 9D). Earlier themes were focused on platelet-rich plasma, coral, BMP-7, and marrow stromal cells.

## 4. Discussion

Recently, Chen et al^[34]^ published a bibliometric analysis on the application of MSCs in bone regeneration, examining literature from 2013 to 2023. Our study reveals significant growth in the field, particularly after 2011, with fluctuations observed between 2021 and 2024. A more comprehensive analysis of literature from the past 2 decades would provide a more accurate reflection of the current state of research.

Additionally, our analysis suggests that many researchers have overlooked the importance of searching titles and abstracts, which has likely resulted in a reduced number of identified articles. In contrast, we conducted bibliometric and visualization analyses of MSC-related studies on bone regeneration using WOSCC, CiteSpace, and R software. Our findings show a consistent increase in publications from 2005 to 2024. Biomaterials emerged as the most published and cited journal in this field. Notably, Liu Y is identified as the most prolific and influential author, having published 167 papers, which have received 5720 citations. Shanghai Jiao Tong University leads in institutional output, and China stands at the forefront in national contributions. Core research strengths are concentrated in institutions and researchers from China, the USA, South Korea, and Germany, reflecting extensive global collaborations in MSC-driven bone regeneration.

### 4.1. Publication outputs and collaboration

Between 2005 and 2024, the annual number of publications in MSCs for bone regeneration has steadily increased, reflecting growing commitment from researchers and significant scientific contributions. Despite this progress, nonhealing bone fractures and nonunions remain major clinical challenges.^[35]^ Among the 9441 published articles, research spans not only medicine but also engineering and technology. Although substantial progress has been made, MSCs have not yet become a standard treatment for bone regeneration, indicating considerable potential for further advancement and justifying continued investment in both resources and manpower. Factors influencing research include healthcare standards, an aging population, and economic and technological advancements, with significant regional variation in MSC research.

China and the USA lead in research output, with Asian countries making up 60% of the top 10, many of which are either high-population nations or those facing rapid aging. China, due to its large and aging population, contributes nearly half of all publications, followed closely by the USA, South Korea, Germany, and Japan. In contrast, India, the most populous country, ranks 10th with only 243 articles, reflecting a substantial gap compared to China and the USA. This disparity may stem from a limited focus on aging demographics^[36]^ and insufficient research funding in India.

China’s research on MSCs began later, with only 4 publications in 2005. However, since 2011, there has been rapid development, with annual publications exceeding 500 articles between 2021 and 2024. This growth coincides with an increase in the number of active researchers, peaking at 3352 in 2024. Among the top 10 institutions, 9 are from China, and all 10 leading authors are affiliated with Chinese institutions. China also leads in funding contributions, followed by the USA and Japan. Iran and India, as developing nations, have made notable contributions, with 354 and 401 funding agencies respectively, though the scale of their output remains small compared to China and the USA.

Despite China’s leading position in publication volume, total contributions, and citations, its average citations per article remain relatively low, ranking second to last among the top 10 countries with an average of 30.16 citations per article, compared to 58.19 for the USA. This suggests that China’s global influence has significant potential for growth, particularly in improving the overall quality of its research. Autologous bone grafting, currently the primary treatment for bone defects, is costly and associated with complications.^[37]^ In China, particularly in the western regions, the high cost of healthcare underscores the urgent need for alternative, cost-effective treatments that minimize complications.

Over the past 2 decades, 1089 journals have published MSC-related studies on bone regeneration. The top 10 journals by publication volume account for 18.8% of the total output, with all having an IF above 3.00, and 5 journals exceeding an IF of 5.00. Leading journals such as *Biomaterials*, *Acta Biomaterialia*, and *Journal of Biomedical Materials Research Part A* are prominent within the biomedical field. Notably, 30% of the top 10 journals by publication volume also rank among the top 10 for co-citations, highlighting their central role in advancing research. For example, the article “*Coupling of Angiogenesis and Osteogenesis by a Specific Vessel Subtype in Bone*” by Anjali P. Kusumbe et al^[38]^ published in *Nature*, demonstrates that certain capillary subtypes facilitate bone regeneration by creating a unique microenvironment for bone vascular growth, linking angiogenesis and osteogenesis.

In an era of increasing global interconnectivity, researchers should leverage available resources and foster collaborations to enhance research efficiency and quality. Cooperation between leading countries, such as China, the USA, and South Korea, remains relatively limited. China primarily collaborates with countries that have low publication output, including Nigeria, Hungary, Northern Ireland, and Yemen, while the USA partners with nations that have higher publication volumes, such as Germany, Sweden, Australia, and Armenia. In China, collaboration is predominantly domestic, with limited international cooperation, particularly among leading institutions and authors. High-output countries and institutions may face limited collaboration opportunities due to overlapping research themes and intensified competition.^[39]^ Top research teams, with clear research directions, often work independently, further contributing to the lack of collaboration among elite groups.

Regarding research growth, Japan has shown the most significant bursts in publication output, followed by France and Israel, while China does not appear among the top 10. The Institut National de la Santé et de la Recherche Médicale (Inserm) ranks first for research output bursts, followed by the University of Bologna and Dankook University. Among the top authors with the strongest bursts, Kim Hae-Won, Li Gang, and Xu Hockin H. K. lead, with only 2 Chinese authors in the top 10. Despite China’s large publication volume, its research output is characterized by a lack of explosive growth, possibly reflecting stable, ongoing academic activity rather than breakthrough innovations, further limited by insufficient international exchanges and collaborations.

### 4.2. Keyword co-word and topic trend analysis

The analysis of keywords and subject terms can reveal trends in the evolution of topics and research hotspots within a specific field. Our findings indicate that the most frequently used terms in relevant studies include osteogenic differentiation, MSCs, bone regeneration, in vitro, proliferation, and BET. In recent years, keywords such as bone metabolism, injectable hydrogel, nanomaterials, mitochondrial function, tendon-bone healing, and antioxidants have become increasingly common. Besides, contemporary subject terms predominantly feature mir-21-5p, mitochondrial function, cell senescence, exosomes, 3D printing, angiogenesis, and EVs. A summary of the main research frontiers and hotspots is presented as follows.

#### 4.2.1. Osteogenic differentiation of MSCs

The osteogenic differentiation of MSCs is a complex process. MSCs can differentiate into both osteoblasts and adipocytes,^[40]^ with these pathways playing a critical role in maintaining bone homeostasis. Several studies have shown that certain lncRNAs regulate osteogenic differentiation in MSCs^[32,41]^ while inhibiting adipogenic differentiation,^[42,43]^ providing promising therapeutic strategies for bone regeneration.

Immune cells are integral to bone healing and regeneration following traumatic injury. At the early stages of a fracture, monocytes are recruited to the injury site,^[44,45]^ where they differentiate into macrophages. These macrophages express and secrete biomediators that recruit and activate MSCs^[46]^ and osteoblasts.^[47]^ Moreover, MSCs promote macrophage transition from classical activation (M1) to alternative activation (M2) through paracrine mechanisms.^[48]^ Immune cells and their cytokines regulate osteoblast formation, primarily through the bone morphogenetic protein (BMP)^[49]^ and Wnt/β-catenin signaling pathways,^[50]^ with macrophages playing a pivotal role in this process.

During osteogenesis, markers such as RUNX2, alkaline phosphatase (ALP), osteopontin (OPN), and osteocalcin (OCN) are commonly used to assess the extent of MSC osteogenic differentiation.^[51]^ Research indicates that macrophages of all phenotypes enhance ALP activity, matrix mineralization, and osteogenic differentiation in MSCs.^[52]^ Tumor necrosis factor-α (TNF-α) and interleukin-1β, secreted by M1 macrophages, influence osteogenic differentiation through distinct signaling mechanisms. While TNF-α promotes endochondral osteogenesis by recruiting osteoclasts and MSCs,^[53]^ elevated levels of TNF-α inhibit the expression of RUNX2, osteoblast-specific transcription factor, ALP, OPN, and OCN via the Wnt/β-catenin pathway, thereby inhibiting osteogenic differentiation.^[54]^

The Wnt/β-catenin pathway plays a crucial role in osteogenesis. Activation of this pathway upregulates multiple Wnt target genes, including sex determining region Y-box 2, nanog homeobox, octamer-binding transcription factor 4, and cyclin D1, which promote MSC proliferation and differentiation.^[55,56]^ Conversely, low levels of TNF-α activate IKK2/NFκB, stimulating BMP2 expression, which enhances RUNX2 and osteoblast-specific transcription factor expression, increases ALP secretion, and promotes matrix mineralization,^[57]^ positively regulating osteogenic differentiation. Similarly, interleukin-1β also promotes osteogenic differentiation by activating the BMP/Smad signaling pathway at low concentrations.^[58]^

Despite the pro-inflammatory effects of M1 macrophages, they may be crucial for MSC recruitment and bone healing. In contrast, M2 macrophages exert a distinctly positive osteogenic influence. They foster a supportive microenvironment for angiogenesis and osteogenesis by activating Smad1 signaling and secreting vascular endothelial growth factor (VEGF) and BMP2. Following angiogenesis, the improved tissue microenvironment further promotes osteogenic differentiation, resulting in a synergistic effect on bone healing.^[52]^ Among the numerous factors involved in bone regeneration, the VEGF and BMP families are particularly significant.^[59]^ Clinical and preclinical studies have demonstrated that the application of VEGF and BMP2 is a critical strategy for treating bone defects and nonunions.^[60,61]^

#### 4.2.2. Frontier research and hotspots of MSCs for bone regeneration

##### 4.2.2.1. Bone metabolism and MSCs in bone regeneration

Bone integrity relies on a balance between bone resorption and formation, collectively known as bone metabolism. This process is regulated by the dynamic interaction between osteoblasts and osteoclasts, which together maintain the equilibrium of bone formation and resorption.^[62–64]^ Osteoblasts, derived from MSCs, form new bone matrix and subsequently mineralize it, leading to mature bone tissue. Bone-specific proteins, such as bone sialoprotein, ALP, OCN, OPN, and type I collagen, are expressed at various stages and regulate osteoblast adhesion and mineralization.^[65]^ Moreover, osteoblasts secrete endocrine factors that modulate glucose and lipid metabolism, balancing osteogenic metabolism with the body’s overall energy demands.^[66]^

Osteoclasts, originating from the monocyte/macrophage lineage, primarily function in bone resorption.^[67]^ They attach to the bone surface and secrete protons and enzymes to degrade the bone matrix.^[68]^ Vacuolar-type H+-ATPase, located at the ruffled border of osteoclasts, creates an acidic environment that facilitates bone resorption.^[69]^ Cathepsin K and matrix metalloproteinases are critical enzymes secreted by osteoclasts involved in this process.^[70]^ The receptor activator of nuclear factor-kappa B ligand (RANKL)/osteoprotegerin axis, central to osteoclast regulation, involves RANKL binding to RANK on osteoclast precursors, stimulating osteoclastogenesis. Osteoprotegerin acts as a decoy receptor, inhibiting this interaction and suppressing bone resorption.^[71]^ Maintaining functional balance between osteoblasts and osteoclasts is vital for quality bone formation, and regulating osteoblast function while inhibiting osteoclast activity is key during bone regeneration.

Various strategies have been developed in BTE to promote this balance. Improving cellular adhesion is achieved by modifying implant surfaces,^[72]^ including increasing roughness, employing nanostructured topographies,^[73]^ and creating biomimetic coatings.^[74]^ Growth factors such as BMP,^[75]^ transforming growth factor-β,^[76]^ and fibroblast growth factors^[77]^ help establish bone tissue homeostasis by modulating osteoblast and osteoclast functions. Metal ions influence protein expression in osteoblasts and osteoclasts by regulating key signaling pathways like MAPK, Wnt, BMP, PI3K/AKT, Nrf, RANKL/RANK, and NF-κB, affecting their proliferation, differentiation, and activity. These strategies hold great promise in promoting bone metabolism regulation and could be powerful tools for treating bone defects.^[78]^

##### 4.2.2.2. Mitochondrial function in osteogenic differentiation

Mitochondria are essential energy producers within cells, regulating cellular functions through continuous fusion and fission.^[79]^ In high-energy-demand organs like the brain, muscles, and bones, healthy mitochondria are crucial for normal function.^[80–82]^ During osteogenic differentiation of bone marrow-derived mesenchymal stem cells (BMSCs), mitochondria activate the oxidative phosphorylation pathway, generating adenosine triphosphate to promote cell mineralization.^[83]^ Mitochondrial acetylation modifications and the activation of the β-catenin signaling pathway further drive osteogenesis.^[84]^ Additionally, osteogenic induction stimulates mitochondrial fission through the CD38/cADPR signaling pathway, promoting the formation of circular structures that enhance mitochondrial secretion and expedite osteogenesis.^[82]^ Mitochondria not only serve as energy providers but also regulate osteogenic differentiation.^[85,86]^ Mitochondrial dysfunction significantly disrupts bone homeostasis and regeneration, impairing adenosine triphosphate synthesis and exacerbating oxidative stress, leading to osteoblast dysfunction, apoptosis, and dedifferentiation.^[87–89]^ Repairing dysfunctional mitochondria may offer a potential therapeutic strategy for bone disease intervention.^[90]^

##### 4.2.2.3. Mitochondrial transfer and bone regeneration

Intercellular mitochondrial transfer, a phenomenon where mitochondria transfer across cellular boundaries, has gained attention as a potential repair strategy.^[91,92]^ Research shows that mitochondria possess dynamic properties, allowing them to integrate into the recipient cell’s mitochondrial network, enhancing cell function.^[93–95]^ In bone regeneration, mitochondrial transfer can modulate the function of BMSCs without affecting the extracellular microenvironment, distinguishing it from treatments involving drugs, growth factors, or biomaterials. This reduces concerns over cytotoxicity or biocompatibility. Studies^[96]^ show that mitochondrial transfer enhances BMSC proliferation, osteogenic potential, and migratory capacity by upregulating aerobic metabolism, promoting in situ repair of bone defects. These findings highlight the role of mitochondrial transfer in cell communication and its significance in bone defect repair.

##### 4.2.2.4. Bone tissue engineering

BTE offers a promising solution for bone regeneration through implantable engineered bone structures that combine osteoblasts, biomaterials, and biomolecules to replace damaged bone.^[97]^ Nanoscale topographical features of BTE materials influence cell adhesion, survival, proliferation, and differentiation.^[98]^ Nanomaterials, with their high surface area, mechanical strength, and ability to induce critical gene expressions, support bone tissue repair and regeneration.^[99]^ Bioactive materials such as hydroxyapatite, tricalcium phosphate, and growth factors support MSC proliferation and differentiation in BTE.^[100,101]^ MSCs are considered the gold standard in BTE,^[102]^ with scaffold-free methods like MSC aggregates^[103]^ and cell sheets^[104]^ and scaffold-based methods using hydrogels,^[105]^ decellularized scaffolds,^[106]^ and technologies such as 3D bioprinting.^[107]^ Clinical trials have evaluated the feasibility of using patient-specific or allogeneic stem cell-derived biomaterial scaffolds for bone repair.^[108,109]^ Despite significant progress, challenges remain before widespread clinical application, including small sample sizes and design limitations in current studies. Future large-scale studies, improved experimental designs, and standardization of MSC culture and scaffold fabrication processes are essential to strengthen evidence for the clinical use of MSC-based BTE.^[110]^

#### 4.2.3. Current status and future perspectives of MSCs in promoting bone regeneration

##### 4.2.3.1. Gene therapies and exosomes in bone regeneration

Recent advances in gene therapies, particularly involving miRNAs, have shown promise for bone repair due to their simplicity and cost-effectiveness.^[111,112]^ miR-21-5p, a noncoding RNA, is widely involved in osteogenesis and angiogenesis.^[113]^ Increasing evidence suggests that miR-21-5p plays a crucial role in various physiological and pathological processes, including stem cell senescence, proliferation, osteogenic differentiation, and angiogenesis. Its expression is positively correlated with bone metabolism,^[114]^ and it enhances osteogenesis-related gene expression by regulating mitochondrial fusion.^[115]^ The NOTCH signaling pathway is critical in the occurrence and progression of bone defects, playing a key role in angiogenesis.^[116]^ Vascular endothelial growth factor A (VEGFA), regulated by the NOTCH pathway, promotes endothelial cell proliferation, migration, and angiogenesis.^[117]^ Additionally, DLL4, a crucial receptor in the NOTCH1 pathway, contributes to angiogenesis.^[118]^ Studies have shown that miR-21-5p promotes angiogenesis of endothelial progenitor cells by regulating the NOTCH1/DLL4/VEGFA pathway, accelerating bone repair.^[119]^ Furthermore, miR-21-5p activates the PI3K/AKT pathway, inhibits its target gene SPRY1, and induces the expression of hypoxia-inducible factor-1α and VEGFA, promoting the proliferation, migration, and angiogenesis of human umbilical vein endothelial cells.^[120]^ These findings highlight miR-21-5p’s potential in bone defect treatment and as a therapeutic strategy for bone repair.

Exosomes, EVs secreted by various cell types including MSCs, tumor cells, macrophages, and dendritic cells,^[121]^ play a pivotal role in intercellular communication by transmitting signals to trigger biological responses in recipient cells.^[122]^ Exosomes regulate target cell functions through their bioactive components, including microRNAs, lncRNAs, proteins, polysaccharides, and lipids. MSC-based therapies, particularly via exosome secretion, have shown promise in bone regeneration by modulating macrophage polarization, promoting angiogenesis, and inhibiting bone resorption.^[123,124]^ Studies have demonstrated that MSC-derived exosomes enhance osteogenesis through the BMP2/Runx2 signaling pathway, especially under mechanical stimulation.

EVs, a type of exosome, are nanoscale lipid particles containing nucleic acids, proteins, and bioactive molecules.^[125–127]^ Compared to other nanodelivery systems, EVs exhibit superior biocompatibility, low immunogenicity, and high stability, overcoming challenges faced by synthetic nanomaterials in clinical applications.^[126,128,129]^ EV-based therapies show considerable regenerative potential^[130,131]^ in repairing large bone defects and have advantages over traditional cell-based therapies by addressing issues such as heterogeneity, immune rejection, and physiological instability.^[132–134]^ EVs play a crucial role in skeletal development, particularly during endochondral ossification.^[135,136]^ Various studies have demonstrated^[131,137–140]^ that EVs from different cell types promote osteogenesis, with miR-25-enriched BMSC-EVs enhancing fracture healing, and BMSC-derived EVs promoting bone formation by upregulating pro-osteogenic microRNAs.

##### 4.2.3.2. 3D printing in bone regeneration

With advancements in biomaterial technology, 3D-printed scaffolds have become a significant application in bone regeneration.^[141]^ Using 3D printing, personalized tissue-engineered bone scaffolds can be precisely fabricated based on CT/MRI images of bone defects, ensuring a perfect fit with the affected area. 3D bioprinting has been successfully applied to create scaffold models for bone defects in various sites, including the maxilla, mandible, craniofacial bones, and long bones.^[142]^ Bone tissue has a complex nanoscale structure that provides excellent mechanical strength, and 3D bioprinting allows for the creation of biomimetic structures with multiple scales, tissue heterogeneity, and intricate microenvironments.^[143]^ To promote osteogenesis, it is essential to ensure mechanical stability and incorporate organic collagen, cell types, and inorganic minerals into 3D scaffolds.

Calcium phosphate scaffolds can be fabricated with controllable nanopores using 3D printing,^[144]^ enhancing the interaction between growth factors and osteoblasts, thus triggering bone regeneration. These scaffolds exhibit osteoconductivity and osteoinductivity,^[145]^ promoting osteoblast activity. The combination of 3D bioprinting with microfluidic devices has demonstrated significant potential, allowing for precise control over microstructure formation.^[146,147]^ Air-assisted 3D printing methods enable the reconstruction of multicellular bone tissue with high resolution and accurate spatial architecture.^[148]^ A multichannel microfluidic chip, combined with airflow from controllable ejection nozzles, induces the formation of helical cell structures, effectively mimicking the physiological characteristics of human bone tissue.^[142]^

##### 4.2.3.3. MSC aging and regenerative potential

MSCs have been extensively studied in preclinical and clinical trials for bone regeneration. However, their effectiveness is closely regulated by donor and recipient microenvironmental factors.^[149,150]^ MSC aging leads to reduced proliferation capacity, impaired differentiation potential, increased oxidative stress, DNA repair defects, and telomere shortening.^[151]^ In vitro, donor age and replicative senescence contribute to these issues,^[152]^ while in vivo applications often require MSC expansion ex vivo, which exacerbates replicative senescence and diminishes therapeutic efficacy.^[153]^ Senescent MSCs show fragmented mitochondria and secrete higher levels of IL-6 and IL-8 compared to younger cells, contributing to senescence-induced growth arrest.^[154–156]^

Targeting mitochondrial pathways may offer an effective strategy for improving MSC lifespan and function, given the importance of mitochondrial health in MSC-based regenerative therapies.^[83]^ Regulating the expression of IL-6 and IL-8 in senescent cells could mitigate cellular aging, offering a promising approach to enhance MSC-based bone regeneration.

## 5. Challenge

Despite substantial evidence supporting the efficacy of MSCs in bone regeneration, several limitations hinder their clinical application. First, understanding the fate of MSCs after administration and ensuring the long-term survival of allogeneic cells present significant challenges. Secondly, the infusion of dead cells may adversely affect patient health. Moreover, risks associated with MSC transplantation, including immune rejection, oncogenic potential, and tumor resistance,^[157]^ complicate further development.

Successful bone regeneration also depends on the adequate concentration and number of transplanted MSCs. Conditions such as osteogenesis imperfecta and osteonecrosis often require large cell volumes,^[158,159]^ which can lead to complications at the donor site. Additionally, the purification process of MSCs is critical, as poorly purified cells may exhibit morphological inconsistencies and limited self-renewal capacity, thus compromising their differentiation potential.^[160]^ Finally, while biotissue engineering has proven effective in animal models, translating these successes into clinical applications remains a challenge.^[161,162]^

## 6. Limitations

This study has several limitations. First, while our bibliometric analysis summarized research on MSCs in bone regeneration from January 1, 2005 to December 31, 2024, studies published in 2025 were not included, meaning recent significant findings may have been overlooked. Second, our literature analysis was limited to data from the WOSCC. Although PubMed could offer a broader selection of articles, it does not provide citation metrics, IFs, or Journal Citation Reports. Merging publications from different databases could also lead to the loss of data. Thus, we chose to rely solely on WOSCC, which may limit the comprehensiveness of our findings. Third, our citation count analysis did not account for potential biases from self-citations, and the number of citations is inherently time-dependent. As a result, more recent publications may not have accumulated as many citations as older works.^[163]^

Despite these limitations, our findings provide a valuable overview of the trends in MSC research for bone regeneration.

## 7. Conclusion

The use of MSCs for bone regeneration represents a promising area of research, with a notable increase in the number of publications over the past 20 years, with journals such as *Biomaterials*, *Acta Biomaterialia*, and *Journal of Biomedical Materials Research Part A* demonstrating the greatest interest in this field. The leading author in terms of publication volume is Liu Y from Shanghai Jiao Tong University in China. Notably, both the top author and institution are from China, underscoring the significant contributions of the country in this area, followed by the USA, South Korea, and Germany. Global research collaboration is extensive; however, there is a need to strengthen communication and cooperation among top authors, institutions, and countries. Concurrently, China should enhance its international collaborations. Current research hotspots include bone healing, bone metabolism, nanomaterials, and dynamics, while mitochondrial function, mir-21-5p, cell senescence, exosomes, EVs, and 3D printing represent cutting-edge and emerging topics within the field.

## Author contributions

**Conceptualization:** Liheng Huang, Lei Li.

**Data curation:** Jiayi Zhu, Rongnan Shi, Qi Deng, Jianxiong Wang.

**Formal analysis:** Liheng Huang.

**Funding acquisition:** Fuhua Sun.

**Methodology:** Jiayi Zhu, Rongnan Shi, Qi Deng.

**Software:** Jianxiong Wang.

**Validation:** Jiayi Zhu, Rongnan Shi, Qi Deng, Jianxiong Wang.

**Visualization:** Liheng Huang.

**Writing – original draft:** Liheng Huang, Lei Li, Fei Xing, Yue Yang.

**Writing – review & editing:** Liheng Huang, Lei Li, Fei Xing, Yue Yang, Jianxiong Wang, Fuhua Sun.
